# A randomized, controlled clinical trial: the effect of mindfulness-based cognitive therapy on generalized anxiety disorder among Chinese community patients: protocol for a randomized trial

**DOI:** 10.1186/1471-244X-11-187

**Published:** 2011-11-29

**Authors:** Samuel YS Wong, Winnie WS Mak, Eliza YL Cheung, Candy YM Ling, Wacy WS Lui, WK Tang, Rebecca LP Wong, Herman HM Lo, Stewart Mercer, Helen SW Ma

**Affiliations:** 1 Division of Family Medicine and Primary Health Care, School of Public Health and Primary Care, The Chinese University of Hong Kong, Hong Kong, China; 2 Department of Psychology, The Chinese University of Hong Kong, Hong Kong, China; 3 New Life Psychiatric Rehabilitation Association, Hong Kong, China; 4 Hospital Authority, Hong Kong, China; 5 Department of Psychiatry, The Chinese University of Hong Kong, Hong Kong, China; 6 Department of Social Work and Social Administration, The University of Hong Kong, Hong Kong, China; 7 Section of General Practice and Primary Care, University of Glasgow, Glasgow, UK; 8 Centre of Buddhist Studies, The University of Hong Kong, Hong Kong, China

## Abstract

**Background:**

Research suggests that an eight-week Mindfulness-Based Cognitive Therapy (MBCT) program may be effective in the treatment of generalized anxiety disorders. Our objective is to compare the clinical effectiveness of the MBCT program with a psycho-education programme and usual care in reducing anxiety symptoms in people suffering from generalized anxiety disorder.

**Methods:**

A three armed randomized, controlled clinical trial including 9-month post-treatment follow-up is proposed. Participants screened positive using the Structure Clinical Interview for DSM-IV (SCID) for general anxiety disorder will be recruited from community-based clinics. 228 participants will be randomly allocated to the MBCT program plus usual care, psycho-education program plus usual care or the usual care group. Validated Chinese version of instruments measuring anxiety and worry symptoms, depression, quality of life and health service utilization will be used. Our primary end point is the change of anxiety and worry score (Beck Anxiety Inventory and Penn State Worry Scale) from baseline to the end of intervention. For primary analyses, treatment outcomes will be assessed by ANCOVA, with change in anxiety score as the baseline variable, while the baseline anxiety score and other baseline characteristics that significantly differ between groups will serve as covariates.

**Conclusions:**

This is a first randomized controlled trial that compare the effectiveness of MBCT with an active control, findings will advance current knowledge in the management of GAD and the way that group intervention can be delivered and inform future research.

Unique Trail Number (assigned by Centre for Clinical Trails, Clinical Trials registry, The Chinese University of Hong Kong): CUHK_CCT00267

## Background

Generalized anxiety disorder (GAD) is one of the most common mental health problems seen in the primary care or community setting [1] and is associated with significant disability and high utilization of health services [2]. According to established evidence [3], medication or cognitive behavioral therapy are the first line treatments for its management. However, side - effects and costs associated with medications can be barriers to treatments and individual cognitive behavioral therapy can be expensive and time consuming. In addition, for both medication and cognitive behavioral therapy, persistence of residual symptoms continues to be an issue even for those who respond to these treatments initially [2]. As a result, there is a need to use other treatment that falls between a minimalist and intensive approach that can reduce patients' symptoms and health care utilization.

One recently established intervention is mindfulness based cognitive therapy based on mindfulness meditation, modelled after the mindfulness-based stress reduction (MBSR) programme [4] at the University of Massachusetts. Mindfulness refers to "paying attention in a particular way on purpose, in the present moment and non-judgmentally."[4] For example, a mindful approach to one's inner experience is simply viewing "thoughts as thoughts" as opposed to evaluating certain thoughts as positive or negative.

Mindfulness Based Cognitive Therapy (MBCT) (formerly called attentional control) is a theory driven treatment approach developed by Zindel Segal, Mark Williams and John Teasdale with the aim to discover a cost-effective treatment approach to significantly reduce relapse and recurrence of major depression [5]. Cognitive therapy techniques involved in MBCT include education about mood symptoms, the role of negative thoughts, and how rumination, avoidance, suppression, and struggling with unhelpful cognitions and emotions can perpetuate distress rather then resolve it. MBCT emphasizes acceptance rather than change strategies and offers no training in changing the content of thinking but rather seeing thoughts as thoughts rather than as reflections of reality (meta-cognitive awareness) [6]. Recently, MBCT has been suggested as a therapy for treating anxiety symptoms [7] since training in present moment mindful awareness may provide a useful way of responding for individuals with generalized anxiety.

Although it has been shown in randomized controlled clinical trials that MBCT can reduce the likelihood of relapse in people with recurrent episodes of depression [8,9], the evidence for the effectiveness of MBCT in reducing anxiety symptoms among people with anxiety disorder is less well established. So far, three pilot studies, one that employed mixed methodology and studies with a pre and post intervention design have been conducted.

In an exploratory mixed qualitative and quantitative study, Finucane & Mercer [10] showed that the use of mindfulness based cognitive therapy for patients with active anxiety symptoms in primary care was both acceptable and beneficial to majority of patients in his study and more than half of the patients in the study continued to apply mindfulness techniques 3 months after the course had ended. In a study by Evans et al [11] that employed a pre and post intervention (without control) design, they showed that there were significant reductions in anxiety and depressive symptoms post intervention in patients with generalized anxiety disorders. Recently, Yook et al [12] showed that there were significant improvements in Pittsburgh Sleep Quality Index, Penn State Worry Questionnaire, Ruminative Response Scale, Hamilton Anxiety Rating Scale and Hamilton Depression Rating Scale scores at the end of the 8-week program when compared to scores at baseline. However, all these studies were pilot studies with small sample size that ranges from 11-22 participants and with no control group which makes it hard to draw conclusion on the effectiveness of MBCT in treating anxiety symptoms.

## Research objectives

### Primary Objectives

The primary objective of the study is to evaluate the superior effects of MBCT in reducing anxiety symptoms in patients with generalized anxiety disorder from the community and primary care at 9 months when compared to Psycho-Education Group using cognitive behavioural therapy and Usual Care (UG) groups.

### Secondary objectives

The secondary objectives of this study are to evaluate the effects of MBCT on:

• Patients' reduction of worry symptoms post intervention at 9 months

• Patients' reduction of depressive symptoms post intervention at 9 months

• Patients' reduction of the use of psychiatric medication post intervention at 9 months

• Patients' increase in quality of life post intervention at 9 months

• Patients' reduction in use of health service post intervention at 9 months

• Patients' reduction in leave of absence from work post intervention at 9 months

## Methods and Design

The current study protocol was approved by the Joint Chinese University of Hong Kong and New Territories East Cluster (CUHK-NTEC) Clinical Research Ethics Committee (CREC).

This study is a randomized controlled trial with three study arms: the MBCT programme led by trained MBCT instructors, Psycho-Education Group (PEG) using CBT principles led by clinical psychologist, and usual care (UC) which will be offered MBCT at the end of the study for ethical reasons. The MBCT and group PEG consist of eight 2-hour weekly sessions. Outcome measures of all participants will be collected at similar time points (baseline, immediately post intervention, and at 3, 6 and 9 months post intervention). The previous studies that evaluated the effectiveness of MBCT for anxiety did not employed a control group; the inclusion of comparison groups, a usual care group and PEG, will be important in evaluating the benefits of MBCT.

Three instructors with at least two-year experience in teaching MBSR or MBCT will lead the MBCT groups while three clinical psychologists with at least master degree in clinical psychology will be hired for conducting the PEG.

### Participants

Entry criteria for the current proposed study will include all of the followings:

1) being 21-65 years of age; 2) having, at baseline assessment, a DSM-IV TR principal diagnosis of generalized anxiety disorder on SCID (Structured Clinical Interview for DSM-IV) [13] and a score of 19 or above using the Chinese version of the Beck Anxiety Inventory [14]; 3) can understand Cantonese; 4) are willing to attend either the mindfulness based cognitive therapy program or the group cognitive behavioural therapy; 5) if patient is on medication for his/her condition, he or she should be on stable doses of medication for 3 months before starting treatment.

Exclusion criteria will include any one of the followings: 1) illiterate subjects as they will not be able to complete the homework diary; 2) psychiatric and medical comorbidities that are potentially life threatening (i.e. psychosis, suicidal ideation, terminal medical illness) or those expected to severely limit patient participation or adherence (e.g. psychosis, current substance abuse, dementia, pregnancy); 3) those who are currently seeing a cognitive behavioural therapist or psychotherapists/counsellors.

Patients will be recruited from: 1) referral from doctors who work in the General Outpatient Clinics (GOPC) of the New Territories East Cluster of the Hospital Authority and who are interested in this study; 2) review of those who have been referred to be seen by a psychiatrist for anxiety symptoms and who are on the wait list to be seen for at least 12 months (currently there are more than 500 patients in this category with a majority stating that their major complaint is anxiety);. 3) a website set up by the University that provide health-related information which is available to general public.

All interested subjects will be screened on phone by a trained research assistant to determine eligibility in accordance to the pre-set inclusion and exclusion criteria. Those eligible subjects will then be scheduled to have a diagnostic interview with a family physician; the family physician will further confirm the eligibly with the Structured Clinical Interview for DSM-IV SCID [13]. Both the family physician and the research assistant are trained to use the DSM-IV SCID with a psychiatrist. All eligible participants will be seen by the principal investigator (PI) 1-4 weeks before the start of the interventions to further explain the study, to confirm eligibility, and provide informed consent.

A statistician who is not involved in any part of the study independently randomized participants by using a predetermined random table generated by Microsoft Excel 2002. These numbers will not be decoded until the intervention group is assigned.

The allocation is concealed from the researchers, who carries out the baseline assessment or recorded the data, and the statistician who carried out the analysis. The allocation is unknown to the participants until the first appointment.

The research study has been approved by the Joint Chinese University of Hong Kong-New Territory East Cluster (CUHK-NTEC) Ethics Committee.

### Mindfulness Based Cognitive Therapy (MBCT) plus usual care - treatment arm

After an initial individual orientation session, the MBCT program will be delivered by an instructor who has been trained in mindfulness based cognitive therapy and who has more than 2 years of training experience in MBCT. One programme of 18 participants will be conducted for 8 weeks with 2 hours group training sessions. The programme will include daily homework exercises which will include guided (taped) or unguided awareness exercises directed at increasing moment by moment nonjudgmental awareness of bodily sensations, thoughts, and feelings together with exercises designed to integrate application of awareness skills into daily life. Key themes of MBCT include empowerment of participants and a focus on awareness and acceptance of experience in the moment. Participants are helped to develop a "decentred" perspective on thoughts and feelings, in which these are viewed as passing events in the mind.

### Psycho-Education Programme based on cognitive behavioural therapy principle - active control arm

The Psycho-Education Programme is designed to be comparable to MBCT in level of structure, therapist's contact and attention, with participants required to adhere to an agenda during sessions with homework assignments of similar duration. It will consist of a series of 8 weekly 2-hour sessions. The sessions are based on White's book on Treating Anxiety and Stress [15]. All instructors will be qualified clinical psychologists with training in cognitive behavioral therapy.

### Usual care control (UC)

Usual care refers to primary or community care used by participants. In Hong Kong, the majority of primary care is provided by the private sector with around 20% provided by public outpatient clinics. In general, patients who use public outpatient clinics tend to be older, with chronic conditions and who are from a lower socio-economic status.

As the consultation time is usually short (usually one doctor sees about 40-45 patients in one morning), they may be lack of time to deal with patients who present with emotional problems. As a result, usual care can mean minimal care especially if the patients are from lower socio-economic status. The use of this control is to test the effectiveness of MBCT against minimal intervention. All controls will be offered MBCT after the end of the study for ethical reasons.

Measures at baseline and each follow-up: Subjects' demographic data including age, sex, marital status, education status, personal monthly income, religious belief, and all other outcome measures (described below), use of medication (including psychotropics), and experience with mindfulness and yoga, together with medical history including psychiatric history will be collected by a trained interviewer. After the baseline visit, subjects will be randomized as described above. The fidelity of the intervention will be ensured and monitored by a random review of audiotapes that are recorded during all sessions.

## Outcome measures

These will be measured at baseline and at 8 weeks (end of intervention) and will be repeated at 3 months and 6 months and 9 months after intervention.

### Primary outcome measures

Anxiety symptoms will be measured using the validated Chinese version Penn State Worry Questionnaire [16] and the Chinese version Beck Anxiety Inventory [17]. The use of two validated questionnaire is to improve validity of findings. The Penn State Worry Questionnaire is a measure of worry phenomena and has been demonstrated valid in cross-cultural populations. In a study that examined the factor structure and psychometric properties of the Chinese version of PSWQ, this scale has been shown to have good internal consistency, and both convergent and discriminate validity [18]. The Beck Anxiety Inventory (BAI) is the measure most frequently used to assess the construct of anxiety. It is a popular measure due to its efficient, cost-effective and easy to administer, complete and interpret with good reliability and internal consistency [17].

### Secondary outcome measures

Depressive symptoms will be measured by the validated Chinese version of the Centre for Epidemiological Studies-Depression Scale (CES-D) [19]. Health related quality of life will be measured by the validated Chinese version Medical Outcomes Study Short-Form Health Survey (SF-12) [20]. The SF-12 is a 12-item survey that reports health-related quality of life, including both physical and mental functioning and well-being. It has shown to be a valid measure for measuring health related quality of life in local Chinese population.

"Mindfulness" will be measured by the Five Facet Mindfulness Questionnaire [21].

Health service utilization and medication use: Utilization of health services including visits to primary care doctors (both private and public) and hospitalizations and the number of days of absence from work attributable to anxiety will be recorded at 4 weekly intervals by the trained research assistant using telephone. The frequency and duration of practice of both MBSR and PEG in each group will be documented by a Weekly Homework Form that will be collected each week during class and at 3 month interval until the end of 9 month post intervention. Moreover, medication use including changes in medication will be monitored at 3 month interval. Response rate (enrolment of subjects), retention rate (number and proportion attended each class) of the MBCT and the psycho-education group will be documented.

### Data and statistical analysis

Baseline characteristics of the three groups will be compared by ANOVA test for continuous variables and chi-square test for categorical variables. The baseline factors will include age, sex, medication status, psychological and lifestyle characteristics. Our primary end point is the change of anxiety and worry score (BAI and Penn State Worry Scale) from baseline to the end of intervention. Secondary outcomes included the change in scores of CES-D and SF-12. For primary analyses, treatment outcomes will be assessed by ANCOVA, with change in anxiety score as the baseline variable, while the baseline anxiety score and other baseline characteristics that significantly difference between groups will serve as covariates. A two-sided P value of 0.05 or less will be considered be statistically significant. For secondary analyses, the three groups will be simultaneously taken into the analysis of variance model for repeated measures. The baseline measurement of anxiety symptoms and any significant secondary outcome variables will be entered into the model as a covariate as they are considered to be confounders. Analysis will also be performed on an "intention to treat" analysis.

Since there have been no studies on MBCT with an active control that control for contact time and attention, we need to use the only available study on GAD with an active control by Wetherell et al [22] for sample size calculation. The null hypothesis for this study is that there will be no improvement in the outcome measures in the MBCT group relative to other groups and we will test this hypothesis using a one-sided analysis.

Assuming that the average change of mean score of BAI of patients in MBCT is 5.6 (7.4), in the discussion group is 2.0 (7.4) and that in usual care control is 1.1(7.4) with a 1-sided type I error of 5% and 80% power to detect statistical significant differences between the MBCT and psycho-education group as well as MBCT and usual care control, the required sample size is 76 subjects in each group. We set our enrolment target at 228 subjects.

## Discussion

This will be the first study conducted to investigate the usefulness of MBCT using a control group in the treatment of anxiety symptoms in those with generalized anxiety disorder. One of the key strengths of this proposed study when compared to previous studies in this field is the use of an active control group since most of the previous studies have only employed a pre-post design or lack of an active controls.

Since the active control group is conducted by psychologists using CBT principles, which is the current recommended low intensity intervention by the National Institute of Clinical Excellence (NICE) for patients with GAD who suffer from milder symptoms of anxiety in the UK, the results of this trial will advance current knowledge in the management of GAD and the way that group intervention can be delivered and inform future research. One of the advantages of MBCT is the potential for having larger number of participants when compared to normal CBT group (range from 6-8). In this study, we plan to have larger group size than group size previously adopted by previous investigators of MBCT trial.

If MBCT is shown to be effective in reducing participants' symptoms, further economic analysis can be conducted to further evaluate the cost-effectiveness of this intervention. Moreover, it can be further disseminated in various primary and community settings for helping patients with GAD in the community.

## Limitations

Participants who volunteer for the study could be biased towards non-pharmacological treatment and those who are assigned to the usual care group may be disappointed and drop out from the study. Although we will offer MBCT at the end of the study, the attrition rate for this group of participants is likely to be higher. Further analysis of the demo-sociographic characteristics of pateints who enrolled in our study will further inform the generalizability of our findings.

## Competing interests

The authors declare that they have no competing interests.

## Authors' contributions

SYSW conceived of the study, and participated in its design and coordination, as well as the drafting of the manuscript. WWSM participated in its design and analysis, as well as revising the manuscript. EYLC participated in its design, as well as the revision of the manuscript. She also participated in the conduct of intervention. CYML participated in the coordination, as well as the revision of the manuscript. She also participated in the drafting of the protocol for the intervention. WWSL participated in the coordination, as well as the revision of the manuscript. She also participated in the conduct of intervention. WKT participated in the study design, as well as data collection and interpretation of data and revising the manuscript critically for intellectual content. RLPW participated in the coordination, management of data collection as well as manuscript revision. HHML participated in the design, as well as the conduct of the MBCT intervention, as contributed to intellectual content of the manuscript. SM contributed to the design of the study, as well as interpretation of data and analysis plan, as well as revision of the manuscript. HSWM contributed to the design, as well as analysis plan and interpretation of data. She also contributed to the revision of the manuscript. All authors read and approved the final manuscript.

## Pre-publication history

The pre-publication history for this paper can be accessed here:

http://www.biomedcentral.com/1471-244X/11/187/prepub

## References

[B1] Nisenson LG Pepper CM Schwenk TL Coyne JC The nature and prevalence of anxiety disorders in primary care Gen Hosp Psychiatr 1998 20 21 28 10.1016/S0163-8343(97)00096-0 9506251

[B2] Simon G Ormel J VonKorff M Barlow W Health care costs associated with depressive and anxiety disorders in primary care Am J Psychiatry 1995 152 353 357 10.1176/ajp.152.3.352 7864259

[B3] National Institute of Clinical Evidence Management of anxiety (panic disorder, with or without agoraphobia, and generalised anxiety disorder) in adults in primary, secondary and community care NICE Clinical Guideline 22, April 2007 20945576

[B4] Kabat-Zinn J Full catastrophe living: using the wisdom of your body and mind to face stress, pain, and illness 1990 New York: Delacourt

[B5] Segal ZV William JM Teasdale JD Mindfulness-based cognitive therapy for depression. A new approach to preventing relapse 2002 New York: Guilford Press

[B6] Roemer L Orsillo SM Expanding our conceptualization of and treatment of generalized anxiety disorder: intergrating mindfulness/acceptance based approaches with existing cognitive-behavioral models Clin Psychol Sci Prac 2002 9 54 68 10.1093/clipsy.9.1.54

[B7] Astin JA Stress reduction through mindfulness meditation: effects on psychological symptomatology, sense of control and spiritual experiences Psychother Psychosom 1997 66 97 106 10.1159/000289116 9097338

[B8] Teasdale JD Segal ZV Williams JMG Ridgeway VA Soulsby JM Lau M Prevention of relapse/recurrence in major depression by mindfulness-based cognitive therapy J Consult Clin Psychol 2000 68 615 623 10965637 10.1037//0022-006x.68.4.615

[B9] Ma SH Teasdale JD Mindfulness-based cognitive therapy for depression: replication and exploration of differential relapse prevention effects J Consult Clin Psychol 2004 72 31 40 14756612 10.1037/0022-006X.72.1.31

[B10] Finucane A Mercer SW An exploratory mixed methods study of the acceptability and effectiveness of mindfulness-based cognitive therapy for patients with active depression and anxiety in primary care BMC Psychiatry 2006 6 14 10.1186/1471-244X-6-14 16603060 PMC1456957

[B11] Evans S Ferrando S Findler M Stowell C Smart C Haglin D Mindfulness-based cognitive therapy for generalized anxiety disorder J Anxiety Disord 2008 22 716 721 10.1016/j.janxdis.2007.07.005 17765453

[B12] Yook K Lee SH Ryu M Kim KH Choi TK Suh SY Kim YW Kim B Kim MY Kim MJ Usefulness of mindfulness-based cognitive therapy for treating insomnia in patients with anxiety disorder: A pilot study J Nerv Ment Dis 2008 196 501 503 10.1097/NMD.0b013e31817762ac 18552629

[B13] Kam IWK Development of the bilingual (Chinese/English) SCID-I (Structured Clinical Interview for DSM-IV Axis I disorder): a study of its reliability and validity in an in-patient population. Dissertation for Part III Examination of Fellowship 2000 Hong Kong: Hong Kong College of Psychiatrist

[B14] Hyman RT Improving discussion leadership 1980 New York: Teachers College Press

[B15] White J Treating Anxiety and Stress: A group Psycho-educational approach using brief CBT 2000 Chichester: John Wiley & Sons, LTD

[B16] Meyer TJ Miller ML Metzger RL Borkovec TD Development and validation of the Penn State Worry Questionnaire Behav Res Ther 1990 28 487 495 10.1016/0005-7967(90)90135-6 2076086

[B17] Cheng SKW Wong CS Wong KC Chong GHC Wong MTP Chang SSY A study of psychometric properties, normative scores, and factor structure of the Beck Anxiety Inventory - the Chinese version Chinese J Clin Psychol 2002 10 4 6

[B18] Zhong J Wang C Li J Liu J Penn State Worry Questionnaire: structure and psychometric properties of the Chinese version J Zhejiang Univ Sci B 2009 10 211 218 10.1631/jzus.B0820189 19283876 PMC2650031

[B19] Cheung CK Bagley C Validating an American scale in Hong Kong: The Centre of Epidemiological Studies depression scale (CES-D) J Psychol 1998 132 169 186 10.1080/00223989809599157 9529665

[B20] Lam CLK Tse EYY Gandek B Is the standard SF-12 health survey valid and equivalent for a Chinese population Qual Life Res 2005 14 539 547 10.1007/s11136-004-0704-3 15892443

[B21] Baer RA Smith GT Hopkins J Krietemeyer J Toney L Using self-report assessment methods to explore facets of mindfulness Assessment 2006 13 27 45 10.1177/1073191105283504 16443717

[B22] Wetherell JL Gatz M Craske MG Treatment of generalized anxiety disorder in older adults J Consult Clin Psychol 2003 71 31 40 12602423 10.1037//0022-006x.71.1.31

